# New Approaches to Exciting Exergame-Experiences for People with Motor Function Impairments

**DOI:** 10.3390/s17020354

**Published:** 2017-02-12

**Authors:** Martina Eckert, Ignacio Gómez-Martinho, Juan Meneses, José-Fernán Martínez

**Affiliations:** Centro de Investigación en Tecnologías Software y Sistemas Multimedia para la Sostenibilidad (CITSEM), Campus Sur Universidad Politécnica de Madrid (UPM), Madrid 28031, Spain; ignaciogmartinho@yahoo.es (I.G.); juan.meneses@upm.es (J.M.); jf.martinez@upm.es (J.-F.M.)

**Keywords:** exergames, rehabilitation, Kinect, gamification, serious games, physical disability, muscle weakness, middleware, children, adolescents, adaptation

## Abstract

The work presented here suggests new ways to tackle exergames for physical rehabilitation and to improve the players’ immersion and involvement. The primary (but not exclusive) purpose is to increase the motivation of children and adolescents with severe physical impairments, for doing their required exercises while playing. The proposed gaming environment is based on the Kinect sensor and the Blender Game Engine. A middleware has been implemented that efficiently transmits the data from the sensor to the game. Inside the game, different newly proposed mechanisms have been developed to distinguish pure exercise-gestures from other movements used to control the game (e.g., opening a menu). The main contribution is the amplification of weak movements, which allows the physically impaired to have similar gaming experiences as the average population. To test the feasibility of the proposed methods, four mini-games were implemented and tested by a group of 11 volunteers with different disabilities, most of them bound to a wheelchair. Their performance has also been compared to that of a healthy control group. Results are generally positive and motivating, although there is much to do to improve the functionalities. There is a major demand for applications that help to include disabled people in society and to improve their life conditions. This work will contribute towards providing them with more fun during exercise.

## 1. Introduction

For a number of years, the possibility of applying serious games for rehabilitation purposes has been thoroughly investigated [1,2,3,4,5,6,7,8,9,10,11,12,13,14,15,16,17,18,19,20,21,22,23,24,25,26,27,28]. It is often claimed that serious games reduce health system costs and efforts as they enable in-home rehabilitation without loss of medical monitoring, and in so doing provide an additional fun factor for patients [22,23,24]. Multiple reviews have summarized the very powerful contributions and reveal that the systems are generally evaluated as feasible, but no state of general applicability has yet been reached [2,3,5,7,11,13].

Most studies are quite specialised and tend to cover the same groups of largely elderly patients (e.g., stroke and Parkinson’s), which do not constitute a credible target group per se for gaming among the population. In addition, the impression is that the same functionalities are being tested repeatedly, without any evolution. Above all, other groups like children and adolescents with chronic diseases are rarely addressed, even though they are an excellent target group and would probably benefit greatly from using exergames as they need to move like any other child but are mostly limited to performing their exercises with a physiotherapist. This is generally boring, time-consuming and prevents them from playing with friends during this time. If instead they could play games involving physical exercises, without it feeling like rehabilitation, due to proper immersion and motivation, they would possibly need fewer sessions with the therapist, which may in turn improve their social life. Commercially available games would be good enough for many children with physical disabilities, if only they were configurable and adaptive to their potential and needs. Remote controls (RC) are typically not sufficiently configurable (button functions cannot be changed or the RC cannot be used with one hand) and are only made for hands (why not for feet or the mouth?) Some RCs are not sufficiently precise in detection, and so the user ends up tired and loses motivation. Motion capture devices like the Kinect sensor seem to provide better prerequisites for exergaming purposes but feature important limitations too, (e.g., detection of fine movements and rotations) such that the needs of many people are still not be covered by commercial solutions.

However, this is not due to the sensors, but rather the software, which lacks configurability for special needs, such as simple adjustments of level difficulties or the option of playing while seated. For the latter, some Kinect games are available [29], but those are hardly the most liked ones, as has been stated by affected users [30]. Therefore, more complex solutions are required to adapt a game to problems like muscle weaknesses (most games require wide or fast movements), spasticity (“strange” movements are not recognized) or the available limbs (for instance configuring a game to be controlled with the feet for players without full hand use).

To fill these gaps, the authors of the work presented here are pursuing the overall aim (as part of a long-term project) of creating an entertaining exergaming environment for adventure games that immerses the players into a virtual world and makes them forget their physical impairments. Knowledge of the gaming industry is applied to create motivating challenges that the users have to solve, which are sufficiently addictive to make the exercises pass to an unconscious plane. The gaming environment is configurable to the user’s potential and requirements. Challenges will be programmable by a therapist and will also adapt themselves to the players automatically real-time, by observing their fatigue or emotional state (lowering the difficulty or switching to more relaxing exercises when needed).

To achieve this goal, a fair amount of preliminary work is required to eliminate barriers present in commercial applications, so as to find suitable methods to configure the game according to the user and to make him feel like any other person during gameplay. The following basic steps of our work are presented in this article:

- The amplification of slight movements, due to muscle weakness, to full movements
- Distinguishing movements needed to control an avatar or a menu from the ones that comprise the exercise
- Proper inclusion of wheelchair users

The gaming environment is built in Blender and combined with a middleware implemented in C# that passes motion information from the Kinect sensor to the game. The middleware is modular and incorporates support for an Android device and a head mounted display (HMD), but in this work, we focus only on the motion capture with the Kinect.

The first basic version of the middleware to perform the data transmission between Kinect and Blender was published in [31]. The most recent modular version was presented in [32], and therefore only a basic outline of it is described in this paper. The focus here is on two completely new approaches, which are motion amplification and the distinction of movements for different purposes: exercise and game control. Furthermore, four mini-games were implemented to perform preliminary tests for proving the feasibility of those functionalities. Results were obtained by testing a group of volunteers suffering from different disabilities together with a control group of children without any physical impairments. To highlight the novelty of our work and to prove its necessity, we give a short review of the state-of-the-art in exergaming for rehabilitation with Kinect in the following subsection.

### 1.1. Related Work

Given that this work is based on the usage of the Kinect motion capture device, related work on systems that apply other devices is not reviewed. The reason is to present an overview of what has been achieved using this sensor in particular for rehabilitative games.

After the Kinect camera was brought onto the market in 2010 and Microsoft released the Software Development Kit (SDK) in 2011, an exponential boom in development took place, with serious contributions in many areas. The vast majority of research belonged to the e-health sector as the Kinect sensor opened new ways to capture human motion in a non-intrusive way. A peak of contributions appeared in 2013, but since then, less has been published each year. After assessing the literature it appears that despite trying out many options, a point of saturation has been reached, without achieving any generally applicable or commercial usage of the Kinect for rehabilitation purposes.

Many usability studies concerning the accuracy of human motion analysis, feasibility and safety prove that the sensor is generally useful for exercises at home, but has certain limitations detecting fine movements and rotations of the hand, foot, wrist, ankle, shoulder and head, which impedes its ad hoc applicability to any kind of exercises needed and hinders precise supervision [1,2,3,4]. The diseases studied have been mostly focused on similar diseases (stroke, Parkinson’s, cerebral palsy (CP), multiple sclerosis (MS)), with stroke patients being the most common (28% [5]). Furthermore, the inclusion criteria generally excluded severely disabled patients, as their movements present detection difficulties [2]. Wheelchairs usually present a special detection problem due to metallic reflections [6,29], although the authors of publications for wheelchair exercises surprisingly fail to highlight this issue [7,8,9,10,33,34]. Gaming environments are not evolving for real usage in health applications, as they…

- …*are limited to non-occluding movements* [2] which restrict applications to a frontal view perspective. Other individuals can interfere negatively by walking into the camera’s view.
- …*lack exactness and may be prone to undesired compensating movements* often used to “cheat” the system [2,11], although [6] is convinced that here the Kinect has advantages over hand-held remote controls, as it can measure movements more exactly.
- …*may have few positive effects when the system’s performance is poor*, resulting in demotivation and frustration when the environment does not respond correctly to movements, lacks feedback, or is boring [2,7,35].
- …*generally lack interesting design and motivating stories*. The authors of [7] published extensive results of their survey of volunteers. Some of them mentioned monotony due to repetitive exercises, the absence of variety and challenges, as well as some frustration when the game did not react as expected.
- …*are mostly addressed to user groups of elderly patients*, which are the main affected group by stroke and Parkinson’s [2,7,12,13,34,35], to the detriment of children and adolescents [4,14,36], who form a group likely to be more easily responsive to gaming.
- …*lack adaptability*, e.g., in [7], the surveyed participants stated that the games were too difficult when they suffered greater impairments, and found them boring when they had high motor functionality. In [37], screens had to be adapted to the range the users could reach with their arms due to muscle weaknesses. Appropriate players are those with a wide range of possible arm movements (spreading), as resting arms near the body impede any true detection of the person [6,29].
- …*lack configurability and well-designed monitoring systems*. The authors of [7] point out especially that it is very important to implement monitoring systems and the possibility to vary and graduate the intensity and duration of practice, in particular, in cases of very repetitive activities and when individuals ignore pain symptoms due to high levels of engagement and motivation.

The newest approaches found are also still tentative, preliminary and drive small sized test series [7,9,15,16]—only commercial applications have been tested exhaustively [1,3,4,11,18,34,38,39]. Design guidelines are appearing but are still very limited [6,19,20]. In [6], the authors present a guide to applications for clinicians, where controller-based and motion-capture-based systems, as well as commercial games, are compared. The general outcome is that they cannot be played by everybody, due to the required wide arm movements, standing position etc. Publications with recommendations for wheelchairs are missing and no work has been found that applies gamification guidelines, which are well known in game designer circles, such as the Octalysis approach [40], to the design of exergames.

Nevertheless, the evaluation of serious games for physical exercises leads to generally positive results. Compared to EyeToy or Wii, the Kinect seems to be the most natural device and offers great promise for creating enjoyable exercises with a low budget [2]. It also has the potential to track specific movements that should be exploited by incorporating complex and adaptive exercises [13]. Here, fine-tuning, user adaptability, monitoring (to prevent over-exertion) and personalized feedback on how movements should be performed, are highly needed elements [2,13].

Hereinafter, some applications found in literature are analyzed regarding the deficiencies mentioned above.

“PhysioMate” [8] is especially addressed to *wheelchair* users. The gaming environment is similar to “JeWheels” [9]: two hand icons have to be moved to grab objects for recycling. To improve balance training and motor coordination, five upper limb movements are captured: weight transfer, rotation, and moving sideways, back and forth. No user tests were performed and no photos of wheelchair users are shown in the publication. As no detection problems inherent to wheelchairs are mentioned, our impression is that this system has not been fully investigated.

Regarding *user-adaptivity*, the European Rehabilitative Way out In Responsive home Environments (REWIRE) project [21] stands out because of its special focus on intelligent systems. Particular publications of the consortium members can be found about “Adaptive Games for Rehabilitation at Home” [22] or “An Intelligent Game Engine for the At-Home Rehabilitation of Stroke Patients” [23]. The authors state the importance of combining effective exercises with compelling games and implemented, similar to us, a set of mini-games that are included using a common theme, in this case, farming. Nevertheless, no results can be found about user tests or the final state achieved in the project, which finished in 2014. In [26], another user-adaptive game is presented that also wraps the exercises in a farming environment: the user is placed on a moving tractor and has to catch apples from the trees that are passing by. The game was tested by patients affected by mild to moderate Parkinson’s disease. It adapts to the player during the play by slowing down and speeding up according to the player’s success.

To enhance the *playing experience* and motivation, various high quality graphic implementations can be found, mostly using Unity 3D [15,25,27], in one case Blender [12]. “Kinect-o-Therapy” [15] is a complex environment consisting of four mini-games, which are gamified versions of the generally prescribed exercises for arms and body. According to the newspaper article found in [24], this system seems to be available on the market, but no updated web page has been found to discover the end results. In the “Apple catcher” [25], the authors present a game, especially designed for patients with hemiplegia as a possible consequence of stroke. The motivation for the player is to reach a score in a limited time, but scenes are neither changing nor have a story. The work in [12] presents a simple game in a medieval setting, aimed at elderly stroke affected users. This is the only contribution found that mentions the use of Blender, specifically for the modelling of the environment. In our opinion, the motivation (capture coins doing the right movements) is not strong enough, as it has nothing to do with the created environment and no story is involved. “ReaKing” [27] (Rehabilitation using Kinect-based Games) is the only system found that uses the Kinect V2. It comprises two different environments with adventure-like scenes, but also lacks a story. Here, the user, represented by a character in third-person view, is walking through different landscapes where, from time to time, objects appear which have to be dodged, while the user performs a walking exercise (on the spot). The motivation is driven by the collection of coins. The “Upper Extremity Rehabilitation Gardening (UERG) Game” presented by [36] is interesting due to the story and user feedback. It has a charming environment to practice every-day movements. User feedback is included nicely into the story, as seeds change colours according to the correctness of movements.

Few systems have been found that are designed for the physical rehabilitation of *disabled children*, and here, most are addressed to CP. The search for publications about gaming systems used for CP lead to many studies that test the functionality and efficiency of using games in general. For instance, [38] presents a preliminary study of the usefulness of commercially available Kinect games. Although the outcomes are positive, the authors state that future studies require the development of **specific** videogames targeted at the treatment of motor symptoms in children with CP. The same conclusion can be drawn from the review presented in [41] concerning the use of commercial video games in rehabilitation: from a total amount of 4240 studies, only 8% were about CP, 25% about weight, 22% about balance and the remaining 45% about stroke, Parkinson’s and aging. The 8% CP studies include [38] and two others that do not present applied games. Only one proposal has been found that directly targets game design: [17] presents an authoring tool based on event recognition, which is applicable to the Kinect and other devices and includes different mini-games, but does not rely directly on the motion capture data. Furthermore, the authors of [36] propose a system to assist patients with spastic diplegia and hemiparesis in their rehabilitation process. Unfortunately, it is actually not a game, as the user is only required to mimic the movements of an avatar. However, the conclusions are positive, stating that impaired children with disorders equivalent to those of the tests’ participants could benefit from the Kinect’s capacities.

The newest proposal found in [28] at the time of writing (January 2017), does not present anything new compared with the formerly presented systems. Here, three exergames evaluate different psychophysical rehabilitation exercises of neurological patients, by means of an avatar on the screen that follows the movements of the patient. A quantitative analysis of the movements is carried out by saving the joint positions of the skeleton in a database, which allows a follow-up of the evolution of the therapy. Here, no real gaming environment is presented; the user is just required to avoid some virtual balls or to step on coloured rectangles. The exercises can be configured by the therapist by changing e.g., the angles for the required arm rising. No seated mode for wheelchair users is supported and only three patients were tested.

### 1.2. Motivation for this Work

Our impression when analysing the state of the art to-date is that development has stagnated. No developments have been found beyond the simple measurement of joint displacements. All approaches, regardless of whether they are implementing simple or more sophisticated gaming environments, are just analysing movements by reading skeleton positions and movements. By doing this, the only basis for exercise design is the mirroring of the patient’s movements and their visualisation by an avatar (a character or a limb shown on the screen). Here, accuracy and speed could be obtained as metrics. The only motivations for the user are to copy the movements of a demonstrating animated character or to move an avatar to reach objects like coins, apples, or kitchen devices. The purpose is always obvious and users notice quite consciously that they are required to do a certain movement. This possibly hurts or at least is very tiring, as it is aimed at an affected limb. In our opinion, this effect itself probably lowers motivation considerably and should be avoided by varying the type of exercise from time to time, inserting relaxing moments and presenting challenges that make the users forget their pain.

Everything should be included in the same gaming environment and make sense, i.e., if one has to reach for objects, this should be for a reason given in the game. Therefore, a realistic storyline is needed and an environment that provides the transition between different scenes. To the best of our knowledge, this kind of environment has not yet been presented, and our purpose is to create a meaningful gaming environment that addresses all mentioned deficiencies. It should be generally accessible by a wide range of users, adaptable to their potential and needs, and above all, during the gameplay, configurable by the therapist, be funny and motivating such that the patient is not aware of doing exercises. To achieve all these objectives, this very ambitious project will need a long time and the involvement of many experts from different areas such as e-health, vision, telematics, machine learning, etc. Therefore, we are still looking for partners and invite interested readers to contact us.

In the initial study presented here, we report the first set of results about a possible method to achieve this, where users with physical limitations can have the same experiences as others inside a virtual environment, which is an indispensable basis to provide full immersion. Section 2 explains the proposed environment and new approaches to handle game motion control. In Section 3, the results of our feasibility tests are presented. Section 4 closes the paper with some conclusions and an outline of our planned future work.

## 2. The Proposed Gaming Environment

The current section is divided into two sub-sections. The first part describes the technical realization of communication between the Blender environment, Kinect and other devices, as well as the amplification of weak movements, which is the main objective of this work. This part of the work was only briefly introduced in [32]. The second sub-section presents the four mini-games, which were implemented to test the functionality of the amplification and two special game control elements.

### 2.1. Technical Realization

The way to pass data from any input device to Blender has to be solved with the help of a middleware, as Blender itself does not provide any suitable interface. Furthermore, the programming language of Blender is Python [42], and in the case of Kinect, the provided SDK library functions are supported only for C#, C++ and Visual Basic.

The general aim of the proposed framework is to integrate different devices in a modular way, such that any device could be added without the user having a problem installing it. As a proof of concept, and to enhance the user experience, we included a middleware for an Android device and one for the HMD Oculus Rift [43].

**Figure 1:**
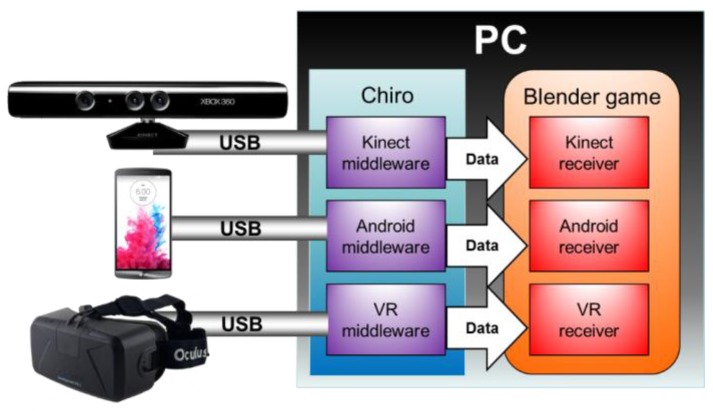
Block diagram of the modular container “Chiro” communicating different peripherals with Blender.

The middleware for all devices are stand-alone programs, each implemented in the language needed for the device, and merged in a software container called “Chiro” as shown in Figure 1. Each middleware individually handles its communication with Blender via USB and a corresponding add-on.

The capture of user movements has been solved with the help of version 1.8 of the Kinect for Windows SDK [44], which provides the *Kinect.Skeleton* class used to obtain the spatial positions of the user’s joints and the rotations of their bones.

While the middleware is in charge of receiving and transmitting the user’s movements, the Blender Game Engine controls the game, based on the data obtained. It is here where the user-specific functionalities have to be implemented, such as configurability according to the user’s abilities, adaptation to the user’s needs and the distinction of different types of movements: those for game control and those for the exercises to perform.

With the help of an add-on, one of four skeleton types designed for this work can be selected to create an avatar. These types provide different functionalities related to the movement—the one that proved to be most useful is one that combines rotation and position data. When receiving motion data from the Kinect, it is copied to the skeleton’s joints. However, this is not enough to create an interactive gaming environment, because the whole of the user’s movements has to be distinguished into pure exercise movements and those required to realize game-control actions as the activation of a menu, for example, to select a weapon, pausing the game or similar. Those movements are normally realized by certain keystrokes or mouse movements and clicks. Exercise-movements will be used to perform actions in the game, such as kicking an enemy or picking up an object and have to be supervised, such that the user performs the actions in the right way. On occasions, the movements would only trigger animations of the avatar, for instance hopping or flying.

**Figure 2:**
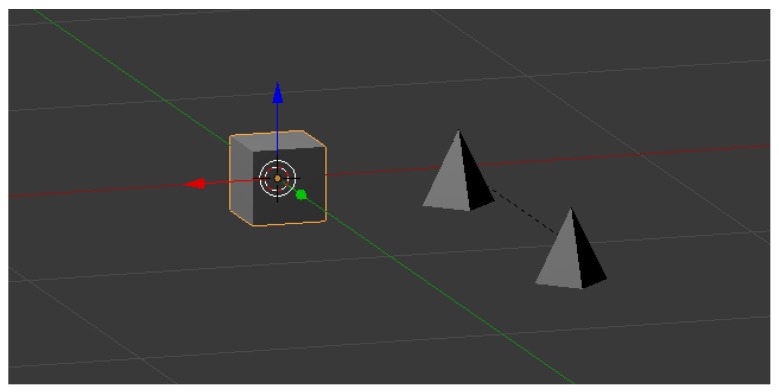
“Accelerometer” (left) and “Rope” gadgets (right).

To control interactions between objects and the avatar’s skeleton easily, two elements called “gadgets” have been created: the “Accelerometer” and the “Rope” (see Figure 2). These gadgets can be used to measure distances and speed by attaching them to objects or body parts and thus avoiding complicated script programming. The “Accelerometer” is a simple cube that incorporates a Python script to measure the speed at which it is moving. The “Rope” has been developed to measure distances. It is composed of two tiny pyramids of the same physical type as the “Accelerometer”, which mark the ends of an imaginary elastic rope. The “Rope” could serve to detect handclapping, a foot stepping on a trap, or the wideness of arm stretching (with the two ends fixed for shoulder and hand).

#### **Motion** **Amplification:**

Our focus is to provide the same sensations and effectiveness in gaming for a special group of users: those who have physical impairments due to illness, injuries, or disabilities. Those can be of many types, e.g., a missing limb, paralysis, or spasticity of body parts or muscle atrophy. To overcome missing limbs, or avoiding those that cannot be used at all, the final application would allow assigning the skeleton joints corresponding to the usable body parts to those of the avatar needed in the game. For example, the arms of the avatar could be played with one arm and a leg.

However, in this work, we focus first on a more challenging problem, which is how to control weak and small movements due to muscle weaknesses, which are present in many rare diseases like muscle dystrophies, muscle atrophies or MS. The aim is to provide the same experiences to affected users as to those without restrictions. If the user is unable to perform a wide or fast movement needed in the game, for example catching or fighting, or unable to raise his or her arms, corresponding movements would appear ridiculous. Nevertheless, if the avatar has to swing a sword powerfully to hurt an enemy, it should do this in exactly the same way as if a person without physical impairments was playing. The diseases mentioned are frequently progressive, which is an additional problem as people are affected psychologically when they realize they can no longer do the same things as before. Here, the gaming environment could also help the user to forget the situation for a moment, if it adapts to the user’s deterioration such that the gaming experience keeps more or less the same.

The following section explains how we resolved restrictions due to muscle weakness by integrating a new object called “Amplifier”. The principle is to detect weak movements and interpret them as being wider than the captured one, based on an initial calibration of the user’s capabilities. The process is divided into two steps: the amplification and its translation to the avatar’s skeleton.

The amplification is achieved linearly by multiplication of the joint positions of the moving limb by a three-dimensional factor in every time instant as expressed in Equation (1):

(1) $$\left[\begin{array}{c} \overset{´}{x}\\ \overset{´}{y}\\ \overset{´}{z} \end{array}\right]=\left(\alpha ,\beta ,\gamma \right)\cdot \left[\begin{array}{c} x\\ y\\ z \end{array}\right]$$

where [x,y,z] is the position corresponding to the user’s limb, α,β, γ the multiplying factors, and $\left[\overset{´}{x},\overset{´}{y},\overset{´}{z}\right]$ the final position of the avatar’s limbs. The factors are obtained during the initial calibration, where the user’s maximum possible movements are captured in all three directions. In this way, a slight movement will be translated into a wider one. Both the factors and the resting point, are configurable as will be explained later on.

**Figure 3:**
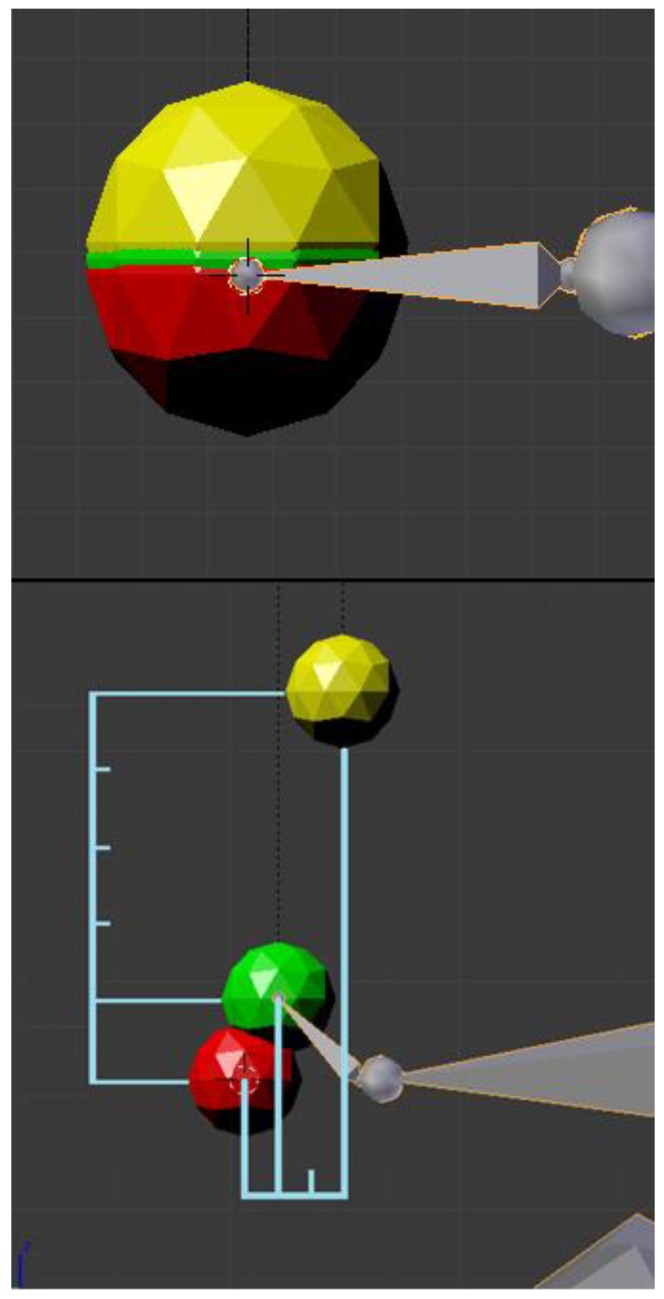
Visualisation of motion amplification. Red sphere = resting position, green sphere = user’s movement, yellow sphere = amplified movement. Upper image: the user’s hand is resting. Lower image: the user raised his hand, which shall correspond to an arm movement.

Figure 3 illustrates how this concept is handled in Blender for the hand as an example case. We added coloured spheres to mark the end of the hand’s bone: the red one represents the position of rest, which is recorded as the origin of every movement; the green one marks the real displacement;, and the yellow sphere is equal to the desired amplified displaced position.

In the upper image, the three spheres coincide, which is the case when the arm is in its resting position. In the lower image, the user did not move his arm he only raised his hand. As can be seen, the yellow sphere has been displaced by a multiple of the displacement of the green one. In this example the amplification corresponds to factor 5 vertically (*z*-axis) and factor 3 horizontally (*y*-axis). The *x*-axis (perpendicular to the 2D viewing plane) has not been contemplated here for simplicity. The blue marker lines have been added to mark the amplifying factors.

The practical implementation of this concept is achieved with the use of an armature as an amplifying object, as it is easy to assign restrictions to the bones to make them follow the movement of a limb. The restrictions also allow the application of multiplying factors to the displacements, but this can only be a value between 0 and 1, which corresponds to an attenuation. Nevertheless, in the presented case, the factors have to be much bigger to cover the range of normal movements, maybe up to 25 depending on the weakness.

**Figure 4:**
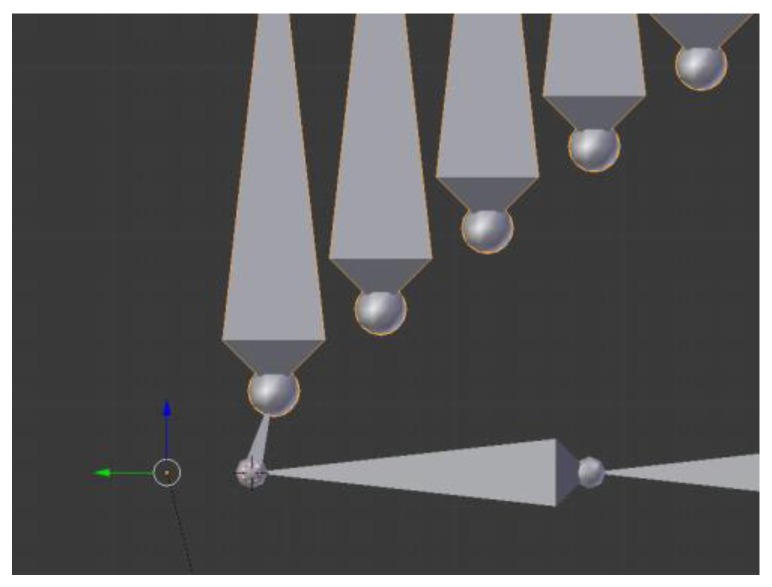
Visualisation of the Amplifier object.

To resolve this matter, a chain of bones has been created, which are concatenated with each of them the “son” of the former one. Each bone copies the local relative position of its “father” to its own local coordinate system, whose origin is at the father’s position. This way, the movement of the first bone is translated to the next one up to the last one in the chain, where it appears as final amplification. To illustrate this process, Figure 4 shows an example for the amplification of a hand motion like the one present in Figure 3. The horizontal bones represent the arm; the small vertical bone is the hand of the user. All vertically oriented bones are copies of the hand, with each one the “son” of the former one. There are 25 bones, each of which represents one amplification factor. Nevertheless, non-integer factors are needed, and a different one in each of the three axes.

To solve this, an additional bone has been added to the chain, which is the one that finally represents the modified displacement *[x,y,z]_m_*. Its father is the very first bone of the chain (the one that copies the limb movement) and it has three restrictions, one for each axis, that make it imitate the position of bone number 25 in the global space. By controlling the value of these factors for the local restrictions *f_res_* between 0 and 1, the final global factor *f* can be determined as follows. First, the local displacement of each of the 25 bones can be expressed as:

(2) $${\left[\begin{array}{c} x\\ y\\ z \end{array}\right]}_{local}={\left(f_{x},f_{y},f_{z}\right)}_{res}\cdot {\left[\begin{array}{c} x\\ y\\ z \end{array}\right]}_{real}\;|\;f_{i_{res}}\in \left\{0\ldots 1\right\},$$

The global displacement factor is, therefore:

(3) $$\left(f_{x},f_{y},f_{z}\right)=25\cdot {\left(f_{x},f_{y},f_{z}\right)}_{res}\;,$$

such that the final displacement is:

(4) $${\left[\begin{array}{c} x\\ y\\ z \end{array}\right]}_{m}=\left(f_{x},f_{y},f_{z}\right)\cdot {\left[\begin{array}{c} x\\ y\\ z \end{array}\right]}_{real}.$$

**Figure 5:**
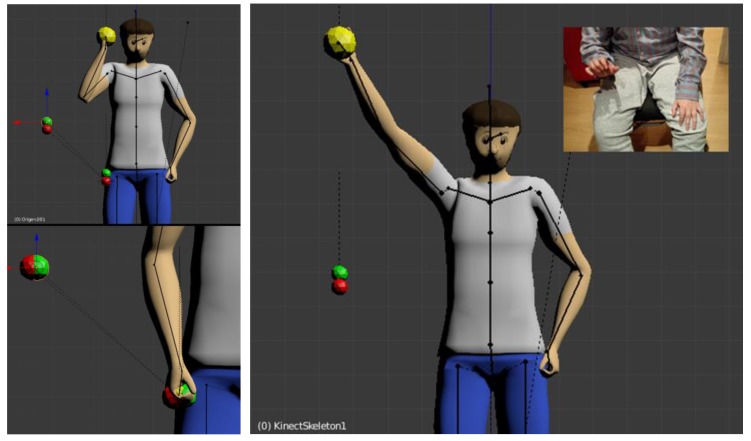
Amplifier with offset and avatar’s movements applying inverse kinematics. Upper left image: the user raises his hand slightly. Lower left image: the user is resting his hand on the armrest of the wheelchair. Right image: the user raises his hand as much he can (he is unable to raise the arm).

The process described up to this point is realized for every outer limb, which means hands and feet. The depending bones (arms and legs) are following through the concept of inverse kinematics. In this way, all bones and joints of the skeleton that are related to one “leading” joint, are following it in a natural manner. Figure 5 illustrates how the real and the amplified movements are interrelated. As before, the red sphere represents the resting position of the player’s hand (as the player here is seated in a wheelchair, it is placed at the height of the avatar’s hip); the green sphere represents the vertical displacement of the player’s hand; and finally, the yellow sphere represents the amplified point, where the avatar’s hand has to be. In the large image on the right, the user is doing his maximum possible movement (see the photo), which is translated to a complete outstretching of the avatar’s arm. On the upper left image, the movement is smaller, such that the hand reaches the shoulder.

The right image reveals one more problem: the resting position of the avatar’s limb. If it were simply copied from the player, in cases like the one presented here, i.e., when the player is seated and the avatar standing, the appearance would not be real, as the avatar would present an arm floating at hip level height when the player is resting. The solution is to implement an offset value that simply displaces the reference point. The result is shown in the lower left image, where the avatar’s hand is resting in the right place.

As each user needs different amplification values and has a different resting position, the need to calibrate the system initially is evident. Therefore, the values are captured with the help of an additional script that registers the resting position and the maximum displacements the user is able to do. To avoid re-calibration in every session, a static skeleton is used (global movement deactivated) that remains static and always looks to the front, wherever the user is. A Python script reads the real positions of the player’s bones and adapts the distance between bones in the skeleton, to his physical appearance. Then, the user is asked to stretch out the arms to all directions as wide as possible. These values are stored in a user-specific file, allowing the skeleton to read them anytime they are needed. To avoid this becoming a tedious process for the user, in a future study, it is planned to be part of the game’s plot.

### 2.2. Preliminary Testing Environment

As a starting point for the future gaming environment, and in order to test the functionalities of the implemented features in case of different disabilities, four mini-games have been created. Currently, each game takes place in a rather boring environment and is represented by a standard avatar, because we wanted to focus only on functionality. Later on, the exercises would take place in different scenes of a gaming environment and the avatar will be replaced by an interesting character.

The implemented exercises should represent a variety of basic movements, which could be tested by wheelchair users with different diseases, so only upper limb movements have been included. The movements have been selected together with a physiotherapist who works with some of the invited volunteers and therefore knows the necessities of those suffering from different rare degenerative diseases. In these cases, the principle aim of rehabilitation is to delay as much as possible the loss of basic functionalities, which are fine and gross motor skills, body and head control. These movements are necessary to perform daily activities and improve quality of life [45,46]. Therefore, exercises that require lateral body and arm movements with shoulder flexion have been implemented: rowing, climbing, hitting, and flying. In the future, further exercises for more precise wrist and forearm movements could be added.

**Figure 6:**
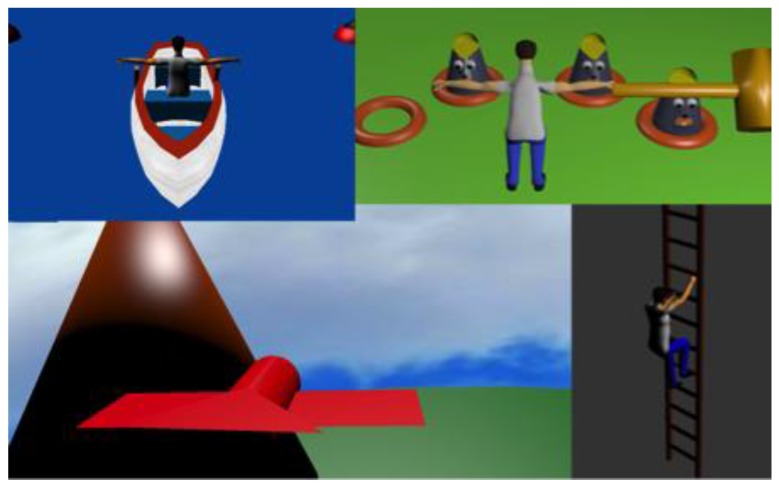
Visualization of the four mini-games (from left to right, top down): “The Boat”, “Whack-a-Mole”, “The Paper-Bird”, and “The Ladder”.

A visualisation of the screens is given in Figure 6. All games were presented to the physiotherapist before performing the trials to assure their innocuousness for the invited test subjects. Test results are presented in Section 3.

#### i. “The Ladder” (Climbing)

The avatar has to climb up to the end of a very long hand ladder and the user has to move his hands up until he reaches the maximum point. Before reaching it, the avatar’s arm is copying the user’s movements (amplified). When the user’s hands get to the maximum position, an animation is executed, the avatar rises its hand and grabs the next bar, releasing the other hand. The feet are completely animated. The detection of the maximum point is realised with help of the “Rope” gadget. During the game, a counter measures the time needed to climb up to the end.

#### ii. “The Boat” (Rowing)

The avatar is seated inside a boat with rows in his hands. Both arms are copying the user’s movements, which should be a simultaneous forward-backward action. The motion is detected with the help of two ropes on each side (one for the forward and one for the backward movement). The boat progresses some meters when all ropes are activated. The aim is to get to the Finish; during the play, the elapsing time appears on the screen.

#### iii. “Whack-a-Mole” (Hitting)

In this game, the avatar uses an enormous hammer, holding it in his right hand to hit some moles that are appearing and disappearing randomly out of four holes around him. The player has to move his right arm up and down in the right direction where a mole appears. If the mole is hit with sufficient speed (measured by the accelerometer gadget), it makes a complaining noise and disappears. The game gives two minutes of time to strike 20 moles, and the number of scored moles is counted.

#### iv. “The Paper-Bird” (Flying)

This is the most sophisticated game regarding the complexity of required movements. A kind of handcrafted bird that is constantly moving forward has to be controlled using the arms (or hands) spread to both sides and the trunk. If the arms are at the same height, the bird is flying straight ahead; if one arm is lower than the other, the bird is turning to that side. Nevertheless, there is no need for the user to spread the arms widely, the hand movements are enough and would be amplified. In addition to the arms (or hands), the back and forth motion of the trunk is captured to control the up-down tilt of the bird. It is conducted through a landscape with mountains, rivers, and yellow rings in the air that the user can pass through. This game has no time limit and goal to accomplish, the only motivation to play is having a good time. No counter was implemented at the time of the trials, so the playing time and the number of rings passed through were noted manually.

## 3. Tests and Results

### 3.1. Testing Environment and Participants

To test these functionalities, the four mini-games were given to two groups of potential users for six days: one group consisted of eight children and three adult women affected by different disabilities as indicated in Table 1. Three of these children had to be excluded from the evaluation, as there were detection issues with their wheelchairs. (Ideally these and future tests should be run on larger sample sizes.) The participants were recruited in Madrid, Spain, at the Neurological Muscular Diseases Association (ASEM) [47] and the Sports Integration Foundation (Fundación TAMBIEN) [48]. The comparative group was formed out of eight children (ages 7–13 years), without any known physical dysfunction.

We are aware that the group of tested persons is too inhomogeneous to obtain meaningful results about the effectiveness of the games for the rehabilitation of certain affectations, but this was not the aim at this stage. Our objectives were:

- Learn about different types of physical restrictions and the corresponding needs for exercises, enduringness, as well as the existence of possible prohibited movements.
- Evaluate the interest of potential users in exergames by finding out their needs and wishes and observing their reactions.
- Check and verify the functionality of the control elements and amplifiers. Find deficiencies and problems to resolve.

### 3.2. Test Procedure

The procedure of the trials has been as follows: only two test subjects were invited each day. First, they (and their parents in the case of children) were briefly introduced to our work and the reasons why we need them for the first trials. Afterwards, a user profile was created as indicated in Section 2, to record the initial (resting) position and to capture the possible motion range. Then, the users played the four mini-games. At the end, they were asked to fill out a short survey to communicate their experience, feelings, and other questions like usual gaming habits or problems they have with games or computer applications in general due to their disability. The main results of the survey are listed at the end of this section; numerical results are analyzed first. The comparative group, consisting of eight children without any known disabilities, were tested all together on a single day. In the same session, all mini-games were also tested with a HMD to compare the experiences. Those results will be presented soon in a separate paper, as they go beyond the scope of the article presented here.

### 3.3. Calibration Results

The very first step was calibrating the amplification for each user, independently of whether they had physical restrictions or not. In this way, the amplifying values for non-affected users were inherently near one, such that maximum user-movements are always translated to maximum avatar-movements.

For users without a wheelchair, the calibration was immediate and did not present any problems, regardless if it was made standing or seated. However, in the case of wheelchair users, the Kinect frequently confused the armrest with the user’s arms or the wheels with the user’s legs, especially in the case of small children. Using the “seated mode” provided by the Kinect, the detection improved for “The Ladder”, but did not work for the other games. In general, our intention is to neglect this mode, because the spine and the legs could be used by many wheelchair users. An interim solution for some children was to sit in a normal chair, but this was not possible for all of them due to their instability. For this reason, no sufficient numerical results could have been obtained for three volunteers (shaded grey in Table 1), who have been excluded from the evaluation.

### 3.4. Blender Mini-Games

The results for the four tested games are presented in Table 2. It contains the average times and scores obtained, indicating also the standard deviation (SD) of each data set. To highlight the differences between groups, the percentage of deviation was calculated and the statistical significance of those values was analyzed. As the available data sets are very small, normal distribution cannot be assured, therefore, the first test was to check if the variances are equal, by performing a Levene test. If the resulting p-value is above 5% (p > 0.05), the variances are considered to be equal, such that analysis of variance (Anova) tests would reveal significant results. The Levene test resulted positive for all data sets, consequently, the t-value (Student’s test) and the F-value (one-way Anova) were calculated. Both are compared for a probability of p = 0.05, which is generally used to decide if two datasets are statistically dependent (t-value > 0.05) or independent (t-value < 0.05). The critical F-value is Fc = 4.6, for the given data sets and according to the F-Table. Values above Fc indicate that the null-hypothesis has to be rejected.

Nevertheless, for “The Boat”, the Levene p-value only holds to 8%, which is quite weak, such that we preferred to check the equivalence of additional data sets with a Kruska-Wallis test, which does not assume a normal distribution. The resulting values are presented as p(chi-sq) in the table and indicate that the Null-hypothesis of data set equality is true if the value is above 5%. For “The Boat”, this test confirms that the populations are different.

**Figure 7:**
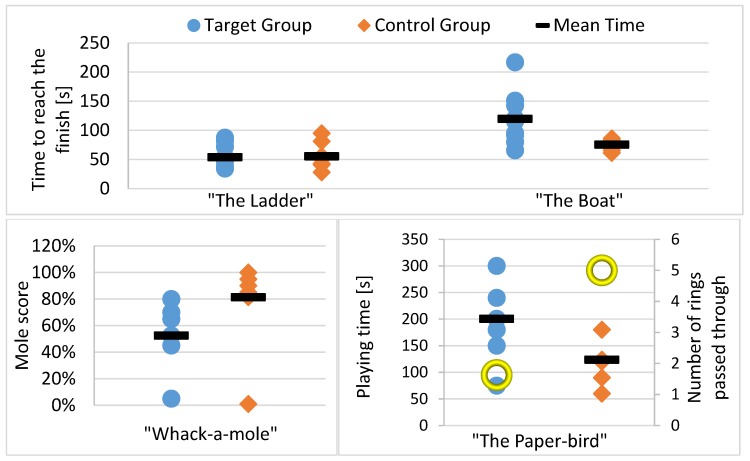
Results obtained for the four tested mini-games.

**Figure 8:**
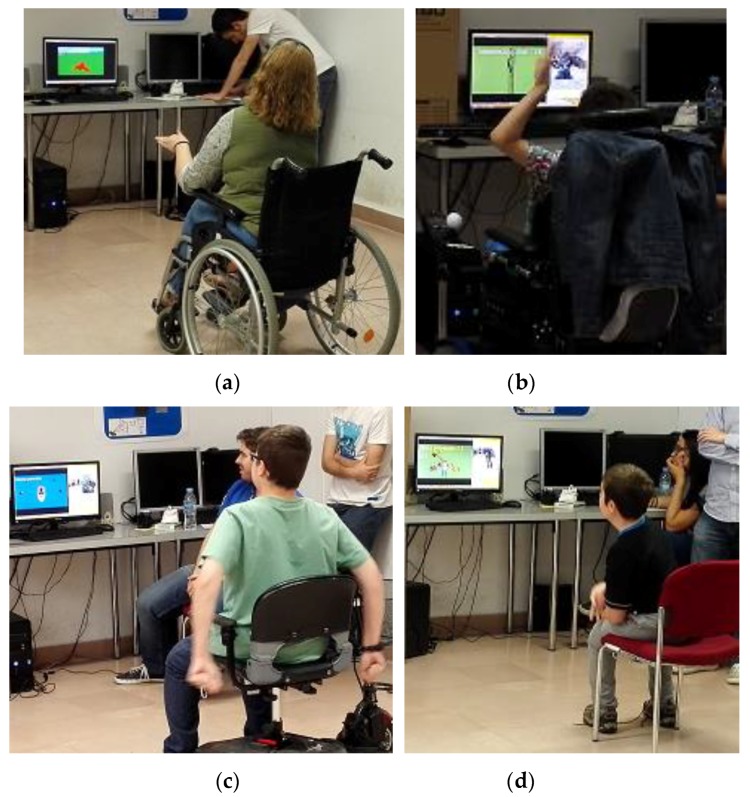
Different scenes while the volunteers were playing. (**a**) “The Paper-Bird”, (**b**) “The Ladder”, (**c**) “The Boat” and (**d**) “Whack-a-Mole”.

The individual results obtained by each subject were visualized in the form of spread plots in Figure 7. Here, the target group (blue dots) and the control group (orange rhombs) can be compared. The average values from Table 2 were added as black bars. In addition, Figure 8 shows some scenes from the trials to illustrate the environment. In the following section, the results and observations from the tests are analyzed individually for each game.

#### i. “The Ladder”

This game requires an up and down movement with the arms or hands, making the avatar climb the stairs until the top was reached. As a measurement of performance, the time to reach the top was clocked. Figure 7a (left side) shows the resulting times for all individuals. In this game, very similar results were obtained for both groups. The t-value and F-value both indicate with a probability of 95% that the Null-hypothesis can be maintained. In other words, the assumption that both groups can play the game with a similar effort is true. This means, that due to the implementation of the motion amplification, the difficulty for both groups is similar, such that this type of exercise could be performed by the disabled together with or competing against others (e.g., in multiple player modes).

In the case of the most severely affected test person, a 15-year-old boy with Duchenne Muscle Dystrophy (ID7 in Table 1), he is not yet able to raise his arms, just his hands, while resting his arms on his legs or on the armrests. Nevertheless, with the help of the amplification, he was able to play the ladder game without problems, and the game reacted in the same way as for any other player. The small photo in Figure 5 shows the movement he made in this game.

#### ii. “The Boat”

This exercise had a similar objective as “The Ladder”: a target line had to be reached rowing, and meanwhile the time was clocked. Nevertheless, the results are not as similar as for “The Ladder”. As can be seen in the graphic (Figure 7a, right plot), the children of the control group performed quite equally and about half of the volunteers of the target group achieved the same time range between 50–100 s. However, the rest had some difficulties, such that the average time of this group is about 45 s longer (60%). The probability values obtained for the variance tests also indicate that both groups are statistically different. The reason is probably that the difficulty of the rowing-movement is quite different for both groups. The purpose was to move the arms forward and backwards, but if the user was unable to do this, the configuration was not sufficiently flexible to capture slightly different movements, such as sideways in a kind of circle. This was the case of ID7—he made the movements in a diagonal manner with hands and wrists while resting the arms on his legs, and, even if the game correctly detected the limits in the front and the back, it responded inconsistently to the sideways motion. As well, the seated position and the wheelchair’s armrests restrict free movement. This probably augmented the difficulties for the target group.

#### iii. “Whack-a-Mole”

In “Whack-a-Mole”, 20 moles were appearing randomly out of their holes and had to be caught in a time range of two minutes. Here, the divergence between both groups was also significant. While the affected group achieved an average score of only 53%, the control group scored over 80%. This could be explained again by the type of movements, which are more difficult than in “The Ladder” and “The Boat”, as they imply reaction time and precision. Here, the functionality of motion amplification is important for the user’s experience (because without amplification, the hammer would barely move in some cases), but only marginal for the execution of the movement, which has to be precise and fast. In addition to weakness and restricted motion ranges, the seated position in a wheelchair apparently hinders the subjects from performing the necessary movements well. Nevertheless, our expectations with regards comparing results between the impaired and unimpaired were not to obtain similar scores, but rather to attain similar feelings during play, as the main goal is to achieve new ways to play exergames with the same fun as others. This was achieved as the t-value and F-value suggest a certain similarity between both groups.

#### iv. “The Paper-Bird”

In “The Paper-Bird”, two values were measured: the total playing time, which was not limited in order to get an idea about the enduringness of the users, and the number of rings passed through, which gives valuable clues about the ability to control different movements simultaneously.

The average playing time of the affected users was much longer than that of the control group, over 3 min vs. 2 min. Whereas several individuals in the control group stated some level of frustration (when passing through the rings was not achieved as quickly as they would have liked), some of the disabled volunteers seemed to be quite motivated just to achieve this. This means that the possibility to pass through the rings was more motivating for them than for the control group and they endured longer. This observation encourages us to continue with this research, as it means that the focus of the game is generally good and just needs to be made a little bit easier, or more flexible in terms of adapting to the user’s potential. It also has to be mentioned that seated and standing users needed different configurations related to the body control functions, so, for this type of game, the configuration has to become much more flexible.

### 3.5. Results of the Survey

After the trials, each volunteer was asked to fill out a questionnaire with two parts: one to obtain general data about the person and their difficulties, their preferences in gaming, former experiences etc. and the other one to evaluate the experience with the four mini-games.

The general questions (Table 3) revealed that most users of the target group are daily and frequent players (73%), who have tried almost all gaming platforms currently available on the market. Surprisingly, 63% of the children in the control group stated only an occasional play, which is probably an understatement due to the presence of their parents. It was also asked for favorite games (we do not reveal the names to avoid advertising), which are above all fighting, motorsports, and football; no adventure games without combat were mentioned. On a 5-point Likert scale, the importance of other aspects like graphical quality, quantity of action or if the game should involve targets to solve, has been evaluated. Here, high importance was assigned to the action and high scores in both groups. However, the most important aspects were the ease of movement (or handling remote controls) for the target group and achieving high scores for the control group. When asked for problems or bad experiences in gaming (only target group), the most frequently mentioned difficulties refer to the remote controls (too much weight, lack of configurability) and that the Kinect did not recognise the wheelchair.

**Figure 9:**
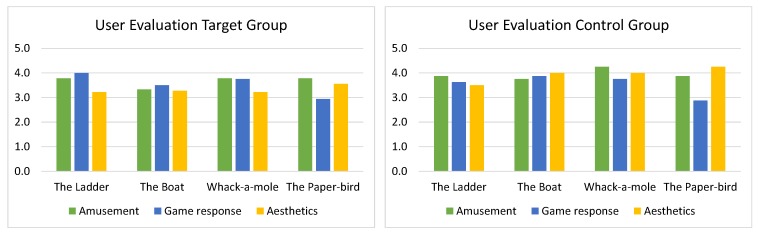
Outcome of the Survey, Part 2—Evaluation of Tested Games.

In the game evaluation part, the users were asked to evaluate the fun-factor, the game response, and the aesthetics of the four mini-games. Figure 9 shows the average points scored on a 5-point Likert scale of those aspects for each game and separated for the target and the control group. The evaluations concur with the observations made during the trials: “The Boat” was the least funny one; “The Ladder” had the best response for the target group, while “The Paper-Bird” had the worst for all. In aesthetical aspects, “The Paper-Bird” wins. For the target group, it is not easy to conclude, which game is considered the funniest, but the control group mostly liked “Whack-a-Mole”, probably due to the action. However, no category was evaluated as either excellent or poor.

### 3.6. Discussion

From a technical point of view, the tests revealed a generally good response to the system. Once the configuration had been achieved, the games were observed to perform correctly, the translation of the movements from Kinect to Blender, the use of auxiliary gadgets (“The Rope” and “The Accelerometer”), and the amplification functionality worked as desired. However, capturing movements was not always successful, depending on the illumination. The Kinect presented problems regarding the detection precision of head, hand, and feet motion, which was sometimes translated to scattering. In addition, the rotation of those articulations cannot be captured with the Kinect V1. Nevertheless, the detection of movements of those body parts would add much value to the exergames, as possibilities for rehabilitation are generally widened. Furthermore, patients suffering from degenerative diseases like muscle dystrophy or atrophy would profit from using their fine motor functions, as normally coarse motor functions are diminishing earlier [45,46]. A further deficiency is the detection problem of individuals in a wheelchair. Here, the arms can be confused with the armrest or the legs with the wheels if the person is small compared to the chair. Hopefully, a future version of the Kinect V2 will improve those functionalities.

Regarding user performance, the control group did not show any problems. “The Ladder”, “Whack-a-Mole,” and “The Boat” were handled with ease and precision, but not without physical effort, as some of the children were sweating, but not complaining. “The Paper-Bird” was the most difficult game to handle − most users found the control system complicated, because arm and body control had to be performed together. In the case of “The Ladder”, with the assistance from the motion amplifier, the volunteers from the target group performed similarly to those of the control group, but this was not the case in the rest of the games. As mentioned before, the reason could be the types of movements, which apart from amplitude require precision and speed. Those factors have to be resolved by additional game mechanics, which would have to be designed in the future. In addition, the wheelchair could be a limiting factor, as it reduces the space in which to move the arms. In general, no user complained about too much effort during play, although they got tired after a while, which is not an undesired effect, as the games are created to perform physical exercises.

The subjective experiences communicated with help of the survey have been positive overall. The amusement factor for both groups was only similar for one game: (“The Ladder”), which is also the one that worked best due to the motion amplification functionality. In the case of “The Boat” and “Whack-a-mole”, the target group enjoyed these less, but “The Paper-bird” more, than the control group, despite the latter needing some improvements. The game response was scored best for “The Ladder”; aesthetics were scored generally lower by the target group. During the tests, positive and motivating side-comments were given about the usefulness of the investigation and that there is a niche to fill. Above all, the parents’ comments got to the heart, and made evident that inclusion is still playing a minor role in our society. However, some limitations of the system have been observed:

The maximum range values obtained during the calibration do not carry information about the motion patterns a player is able to perform. This was noticeable in the case of “The Boat”, where some participants were not able to do the movement as required. Another concern is that, even if users are able to reach the maximum range recorded during the configuration phase, it is not guaranteed that they are able to repeat this movement many times. Here, the game would need sophisticated intelligence to handle variations due to fatigue, such that the player would neither experience frustration, stopping the game, nor over-exertion. A further limitation is that the quality of movements is still not controlled. This is important to avoid the use of compensating movements, which could be adverse in some cases. The games designed so far present different difficulties for playing seated and standing. Therefore, most people in a wheelchair had a reduced motion range and the results of performance were quite different.

Overall, we learned from the observations, numerical and survey results, how the principles of the mini-games should be integrated into the planned full gaming environment. “The Ladder” could be transformed directly to situations where the avatar has to climb a tree or some steep obstacle, such as a hill. The movement will be useable in many situations, also sporadically, such as mounting a horse or tractor or entering a vehicle. The functionality of the rest of the exercises has to be improved and could be used in different scenes as well, e.g., “Whack-a-Mole” could be transformed to construction tasks (hammering nails or chopping wood) and “The Bird” could be flying as well as diving under water.

## 4. Conclusions and Future Work

### 4.1. Conclusions

In this work, preliminary concepts are designed and tested that could build a basis for the implementation of challenging and exciting exergames for everybody. Two important deficiencies have been detected in the field of research into rehabilitation games, which are, on the one hand, the absence of games targeted towards young people with severe disabilities, and on the other hand the implementation of real gaming aspects like storylines, rewards and other types of motivations that could be easily ported from the gaming industry. To combine both, the authors are working on a long-term project, which is the design and creation of an intelligent and user adaptive adventure-like exergame, with the objective of improving the immersive experiences and consequently the motivation for the physically impaired to do exercises. The methods presented here of motion amplification and game control functionalities proved to be feasible and provide a basis to create these kinds of advanced exercise games. The functionality of motion amplification could also be used to create an interface for the disabled that provides access to commercially available games. Currently, the authors are working also on this kind of interface and already presented a preliminary version in [49]. Test results will also be published soon [50].

### 4.2. Future Work

The next step will be to work out a basic gaming environment that includes the tested movements in a challenging story. Here, the first aim will be set on capturing the user in a way that he is motivated to go on in the game to achieve better scores each time, such that daily exercises are assured. Furthermore, the lessons learned about calibration, configuration, etc. will be worked out to find better solutions but, most importantly, the wheelchair distinction has priority. At present, amplification works well, but additional game mechanics are needed to resolve difficulties in performing actions that require speed and precision. Here, the system could provide other types of amplifications to improve the user’s experience.

A medical interface will be created to configure the game and to provide recordings of the user’s movements, performance, and playing history. We would welcome any kind of comment from a medical perspective with respect to requirements and preferences.

The whole platform will be adapted to the Kinect V2 sensor, which provides a higher resolution and more joint data. In addition, the SDK of V2 contains more versatility, above all for facial recognition.

As our group is also working on emotion recognition and facial exercises, those would be included as additional possibilities for exercises in some future version of the full exergame.

The HMD has already been tried out to test different immersive experiences; test results will be presented in a separate publication. The next step here would be to achieve a similar experience with a smartphone used as a VR headset to keep the costs for users low.

A further necessary future study will be the development of intelligent algorithms that assume the role of user adaptiveness during play. With the inclusion of context and emotion awareness concepts, the aim will be to achieve a fully user adaptive game, that reacts to fatigue and evolution of the user by adapting the difficulties of the levels and changing the types of exercises necessary as required. Therefore, close collaboration with clinicians will be needed, in order to determine the best way in which to implement this.

## Figures and Tables

**Table 1:** Overview of volunteers. Subjects 1, 2 and 4 (grey) are not included in the evaluation as their wheelchairs produced detection problems.

Subject ID	Disease	Gender	Age	Wheelchair
1	SMA (Spinal Muscular Atrophy) type 2	male	5	Yes
2	SMA (Spinal Muscular Atrophy) type 2	male	13	Yes
3	CP (Cerebral Palsy)	male	11	Yes
4	CP (Cerebral Palsy)	male	12	Yes
5	Hypotonia	male	12	Yes
6	BMD (Becker Muscular Dystrophy)	male	13	No
7	DMD (Duchenne Muscular Dystrophy)	male	15	Yes
8	DMD (Duchenne Muscular Dystrophy)	male	16	Yes
9	FSH (Facioscapulohumeral Muscular Dystrophy)	female	43	Yes
10	FSH (Facioscapulohumeral Muscular Dystrophy)	female	49	No
11	PPS (Post-polio Syndrome)	female	50	No

**Table 2:** Numerical results and statistical analysis per game (Student t-test, one-way Anova and Kruskal-Wallis test (chi-square)). The critical values obtained for p = 0.05 from the t-table and the F-table are respectively t_c_ = 1.76 and F_c_ = 4.6 respectively. *Null-hypothesis not rejected. Grey values are not significant for the data set tested and are only added for completeness.

Game	“The Ladder”	“The Boat”	“Whack-a-mole”	“The Paper-bird”	
**Parameter**	Average time [s] ± SD		Score ± SD	Av. flying time [s] ± SD	Av. n° rings passed
**Control group**	55.68 ± 21.96	75.64 ± 8.41	81% ± 31%	123.75 ± 38.06	5 ± 3
**Target Group**	53.99 ± 22.44	119.83 ± 48.99	53% ± 22%	200.63 ± 71.52	1.63 ± 1.8
**Difference **	−3%	+59%	−35%	+62%	−67%
**Levene test**	0.93*	0.08	0.58*	0.11*	0.12*
Variances are:	Equal	Diff	Equal	Equal	Equal
**p (t-test)**	0.88*	0.02	0.06*	0.02	0.02
**F-value**	0.02*	6.32	4.10*	6.30	6.52
**p (chi-sq)**	0.56*	0.02	0.01	0.03	0.02
Groups are:	Equal	Diff	Equal	Diff	Diff

**Table 3:** Outcome of the Survey, Part 1—General Evaluation of Video Games. The question “ease to move” referred to the corporal movements to be made with or without remote controls.

	**Daily**	**Frequently**		**Occasionally**		**Never**
Target Group	18%	55%		18%		9%
Control Group	25%	13%		63%		0%
	**Graphics**	Ease to Move*	**Action**	**Online Play**	**Challenges**	**High Scores**
Target Group	4	5	4	4.3	3.8	4.5
Control Group	3.75	3	4.6	3.9	3	5
